# Immersing undergraduate students into research on the metagenomics of the plant rhizosphere: a pedagogical strategy to engage civic-mindedness and retain undergraduates in STEM

**DOI:** 10.3389/fpls.2014.00157

**Published:** 2014-04-30

**Authors:** Erin R. Sanders, Ann M. Hirsch

**Affiliations:** ^1^Department of Microbiology, Immunology and Molecular Genetics, University of California—Los AngelesLos Angeles, CA, USA; ^2^Department of Molecular, Cell and Developmental Biology, University of California—Los AngelesLos Angeles, CA, USA

**Keywords:** undergraduate research, STEM persistence, civic engagement, rhizosphere, genomics

Do you find yourself teaching courses and watching the students' eyes glaze over, clearly disengaged from the class material? Are you concerned that bright young undergraduates spend too much time memorizing esoteric facts from a textbook and regurgitating them on exams rather than motivating themselves to delve deeply into scientific literature, explore the questions and issues confronting modern scientists, and seek out solutions by employing their critical thinking skills and creativity? Have you ever asked your undergraduate students how they would make 100 mL of a 10% sucrose solution and they don't know the answer? Students most certainly encounter this type of quantitative reasoning problem in their Chemistry courses, so are you surprised when transferring this highly practical skill set to analogous problems in a Biology course presents such a challenge to students? If any of these questions provokes a “yes,” you should consider offering a course that ties research and teaching together and at the same time addresses issues that are relevant to real life. By implementing such courses at UCLA, where we focus on global climate change, soil erosion and degradation, increasing numbers of antibiotic resistant bacteria, and non-sustainable farming and forestry practices for a growing human population, we have not only energized our own teaching, but also have inspired our students to become fully engaged in a scientific undertaking to contend with globally-relevant environmental issues confronting twenty-first century society.

## The persistence problem

Engaging undergraduates in authentic research experiences, particularly if this immersion occurs during the first 2 years of college, is a well-documented intervention shown to increase the likelihood of students persisting in science, technology, engineering, and mathematics (STEM) majors and matriculating into post-graduate STEM degree programs (Eagan et al., 2013; Graham et al., 2013, and references therein). Research immersion creates a pathway for us, as faculty, to attract and motivate talented students, including members of groups underrepresented in STEM. By giving students a positive and rewarding research experience, we can sustain or even increase our students' interests in STEM careers (Lopatto, 2007). Retaining a diverse and highly capable student population en route to STEM degrees and vocations is critical to confronting and averting two nationally recognized, interconnected education problems—the impending shortfalls in U.S. college graduation rates of STEM majors (PCAST, 2012) as well as the progressive and disproportionate loss of underrepresented groups at every level of post-secondary education in STEM fields (NRC, 2011; Garrison, 2013).

## Intersection of technology and teaching

Propelled by an era of technological innovation in high throughput DNA sequencing, computational capacity, and bioinformatics, metagenomics has emerged as a multidisciplinary field of research (NRC, 2007) poised with opportunity to captivate our undergraduate students and challenge them to explore questions at the cutting-edge of scientific inquiry. Like genomics, research in metagenomics may involve only a handful of students in our research laboratories or can be incrementally staged and scaled to encompass dozens of students in a course or hundreds of students across the undergraduate curriculum (e.g., Ditty et al., 2010; Jordan et al., 2014). As a learning tool at the interface of biology and technology, metagenomics itself creates experiences for students to use an array of bioinformatics tools, to ask questions that scientists have never been able to consider before, and to develop quantitative competencies and computational skills demanded of a qualified and technologically sophisticated twenty-first century STEM workforce (AAAS, 2011; PCAST, 2012).

## Course-based research experiences at UCLA

Next-generation sequencing technology coupled to advances in computational methods allow us to analyze genomes of the uncultured microbes, a feat that transformed the field of microbiology not much more than a decade ago (Wooley et al., 2010). Microbial metagenomics provides an excellent opportunity for hands-on, experiential learning with cost-effective research projects designed to engage large numbers of undergraduates in authentic, technology-driven, discovery-based research. At UCLA, we have created two courses in which students are immersed in research studies focused on microbial interactions in the rhizosphere of bioenergy-relevant plants and also on plant-growth promoting bacteria (PGPB) isolated from the rhizospheres of plants growing in arid or acid soils. These upper-division courses are part of a larger, interdepartmental laboratory curriculum in Life Sciences that enables hundreds of undergraduates per year to immerse themselves in authentic research experiences as a requirement to fulfill their STEM major (CRLC, 2013).

As participants in this laboratory curriculum, students experience the process of scientific discovery and inquiry as research teams working collaboratively over a 20-week timeframe (i.e., two consecutive 10-week quarters). Each two-quarter series enrolls between 30 and 40 students and may be offered multiple times per year. Students are encouraged to enroll in these courses during their junior year, before they take most of their upper-division electives. This design strategy allows students to acquire experiential context for the more specialized and advanced coursework in their majors. In addition, should student participation in these course-based research experiences inspire some of them to become more interested in research, participation during their third year of college allows enough time for students to pursue an independent research apprenticeship with a faculty mentor during their senior year.

An evaluation of this interdepartmental laboratory program is ongoing. According to self-report surveys given to students enrolled in the research-based courses between 2010 and 2013, students report significant gains in a variety of research skills, with gains for underrepresented minority students (URMs) significantly larger than for non-URM students in half of the categories surveyed (Sanders and Levis-Fitzgerald, unpublished data). Furthermore, when asked to describe their post-baccalaureate plans, a majority of students cited aspirations to pursue scientific careers. These assessment results suggest that the research-based curriculum is an effective intervention in the upper-division courses for sustaining URM student enrollment in STEM courses and for promoting academic success in STEM coursework, thereby addressing major disparities in graduation rates for URM vs. non-URM students (Garrison, 2013).

Students in two of the four research courses in the laboratory program have focused on metagenomics of the rhizosphere, the inspiration for this article. They start off in the field sampling heterogeneous microbial communities from the soil surrounding plant roots, and then proceed to the laboratory where they use both cultivation-based and molecular methods to detect hundreds to thousands of microbial species present in each soil sample (Daniel, 2005). Students form teams comprised of 3–4 people, with each team responsible for the analysis of a different soil sample. Although the cost of next generation sequencing is becoming increasingly more economical, environmental sequencing of the soil microbiome itself is still outside the typical scope and budgetary constraints of undergraduate courses. Instead, our students conduct gene surveys of their soil communities by creating 16S rDNA libraries from metagenomic DNA extracted directly from the soil. Following targeted DNA sequencing on a Roche 454 FLX titanium instrument, the service provider (MR DNA, 2011) supplies each student team with a 16S rRNA gene dataset containing thousands of sequences. These data are taxonomically classified by the service provider into operational taxonomic units (OTU), or bins with smaller numbers of related sequences (97% similarity cutoff for species identity, 95–97% for unclassified species, with the final cutoff for an unclassified phylum at 77%), using a NCBI nucleotide BLAST search against curated databases such as RDP II, Greengenes, and Genbank (Cole et al., 2005; DeSantis et al., 2006; Benson et al., 2013). This analysis provides taxonomic resolution of each OTU from the phylum to genus, and in some cases, species level. Students use these large datasets to evaluate the relative abundance and distribution of OTUs within the broader taxonomic levels. For example, students quantify and graphically present the total number of phyla in a soil sample as slices in a pie chart, with the size of any given slice representing the total number of sequences classified as an OTU in that particular phylum.

This type of metagenomic analysis is routinely done in microbial ecology, generating results that capture granular details of community structure in complex habitats like the soil, and gives students first-hand experience manipulating large datasets characteristic of bioinformatics research. In our courses, these efforts are complimented by PCR amplifying and Sanger sequencing the 16S rRNA gene from a subset of cultivated species. To further explore soil community composition, student teams select a subset of OTUs from the metagenomic analysis that are taxonomically similar to their bacterial isolates for construction of phylogenies used to infer the evolutionary relationships among detected bacterial lineages. So while the metagenomic analysis affords students the opportunity to discover novel sequences derived from genomes of previously undetected microorganisms, obtain finer taxonomic resolution by inferring 16S rRNA gene phylogenies using different techniques, and measure the abundance and distribution of all detectable bacterial lineages in the community, they also can evaluate cultivation bias by comparing sequences from cultured species to the metagenomic gene library. Student discoveries in our courses have included PGPB (Schwartz et al., 2013), cellulase-producers (Attia et al., unpublished data), and several potential diazotrophs (Lee and Hirsch, unpublished data). Student teams identify characteristics of cultured species likely responsible for their plant-growth promoting properties. Because genomes can be used to find specific traits, in subsequent quarters, these microbes (or the soil microbiome itself) may be subjected to whole-genome sequencing. We invite students from courses across the curriculum to help annotate these genomes (or metagenomes), searching for genes correlated to PGPB function.

## Make it relevant and civic-minded

Civic orientation among today's college students is on the decline (Twenge et al., 2012), meaning that students are less mindful of the environment and less interested in taking action to conserve energy or preserve precious natural resources like clean water and fertile farmland. A decline in civic-mindedness goes against the widespread belief that concern about the environment is a top priority among our students. This crisis of green consciousness strongly suggests we need to engage our students in educational activities that help them identify with the natural world and relate to environmental issues. Immersing undergraduates in civic-minded research projects should help students realize how their efforts can stimulate global conservation efforts. Such research experiences, whether in a course or faculty mentor's laboratory, should aspire to invigorate this generation's commitment to more ecofriendly community action.

In our two courses at UCLA, we contextualize the students' investigations of the soil microbiome around civic-minded issues related to the plant rhizosphere. For instance, we devote time talking to students about sustainable agricultural practices: how nitrogen-fixing bacteria can be utilized as biofertilizers (Vessey, 2003), replacing costly chemical fertilizers produced by processes that rely on non-renewable energy sources (Smith, 2002). Student teams then try to cultivate diazotrophs on a suitable enrichment medium, sampling soil from rhizosphere from legumes. In some cases, students may also perform targeted DNA sequencing of genes encoding the nitrogenase enzyme (*nifH*) to verify diazotrophy in addition to the 16S rRNA gene. Other students perform “trap experiments,” looking for microbes that elicit nodule formation and nitrogen fixation on various legumes. So, in addition to learning about soil community diversity, some students gain knowledge about microbial activities in this terrestrial environment through the detection of *nifH* or other metabolic genes whereas others directly work with plants and look for microbes that can help them grow not only via nitrogen fixation, but also through the production of auxin or siderophores or by solubilizing rock phosphate. We also spend time in our courses discussing global energy consumption and the need for developing renewable energy sources such as biofuels. Student teams try to isolate cellulase-producing microbes by screening bacteria they cultured on a medium containing carboxymethylcellulose (Teather and Wood, 1982). Their findings reveal microbes that could potentially enhance the degradation of feedstocks for the production of biofuels. In subsequent courses, other students annotate the genomes of cellulase-producing bacteria or PGPB, searching for the genes responsible for these metabolic functions. These “blue sky” research investigations are highly relevant to the world our students are facing, and it is our job to make sure they become invested in addressing and solving these real world problems–challenges that will take grand solutions and unconventional thinking to resolve.

What better way to address broader impacts on your next grant proposal than to creatively integrate your research and teaching in a way that fosters civic engagement and eco-consciousness, and at the same time supports learning and persistence of all students in STEM? Even more so, what better way for us, as educators, to be involved in major issues confronting these young people who will live in a much more crowded world than we have lived in—a world that will be looking to produce enough food and fuel for the 10 billion of us projected for 2050?

### Conflict of interest statement

The authors declare that the research was conducted in the absence of any commercial or financial relationships that could be construed as a potential conflict of interest.
